# Incorporation of SKI-G-801, a Novel AXL Inhibitor, With Anti-PD-1 Plus Chemotherapy Improves Anti-Tumor Activity and Survival by Enhancing T Cell Immunity

**DOI:** 10.3389/fonc.2022.821391

**Published:** 2022-03-09

**Authors:** Wongeun Lee, Dong Kwon Kim, Chun-Bong Synn, Hee Kyu Lee, Sungho Park, Dong-Sik Jung, Yewon Choi, Jae Hwan Kim, Youngseon Byeon, Young Seob Kim, Seul Lee, Soyeon Lee, Yunjoo Joo, Eun Ji Lee, Mi Ran Yun, Seong Gu Heo, Wookyeom Yang, Ji Eun Jung, Eun Kyung Kim, Jooyeon Park, June Dong Park, Doo Jae Lee, Hyeon-Woo Kim, Sun Min Lim, Min Hee Hong, Beung-Chul Ahn, Jii Bum Lee, Kyoung-Ho Pyo

**Affiliations:** ^1^JEUK Institute for Cancer Research, JEUK Co., Ltd., Gumi-si, South Korea; ^2^Severance Biomedical Science Institute, Yonsei University College of Medicine, Seoul, South Korea; ^3^Department of Discovery Biology, Oscotec Inc., Seongnam, South Korea; ^4^Wide River Institute of Immunology, Seoul National University, Hongcheon, South Korea; ^5^Department of Pediatrics, Seoul National University College of Medicine, Seoul, South Korea; ^6^Division of Medical Oncology, Yonsei Cancer Center, Yonsei University College of Medicine, Seoul, South Korea; ^7^Center for Lung Cancer, National Cancer Center, Goyang-si, South Korea; ^8^Division of Hemato-oncology, Wonju Severance Christian Hospital, Yonsei University Wonju College of Medicine, Wonju, South Korea

**Keywords:** AXL receptor tyrosine kinase, non-small cell lung cancer, chemotherapy, immunotherapy, epithelial-mesenchymal transition

## Abstract

A recently developed treatment strategy for lung cancer that combines immune checkpoint inhibitors with chemotherapy has been applied as a standard treatment for lung adenocarcinoma (LUAD) and lung squamous cell carcinoma (LUSC), and it has improved the outcomes of chemotherapy. Maintenance treatment with anti-PD-1 antibody (aPD-1) enhances the effect of immunochemical combination therapy and improves therapeutic efficacy, which contributes toward a significant improvement in patient survival rates. The AXL receptor tyrosine kinase (AXL), which is expressed in tumor cells, plays an essential role in the resistance of cancers to chemotherapy and immunotherapy, and stimulates signaling associated with epithelial-mesenchymal transition (EMT) in metastatic cancer. AXL is thus an attractive target for controlling resistance to anti-tumor therapies. In this study, we examined the effect of AXL inhibitors on immune activation and tumor growth in TC1 and C3PQ mouse tumor models, in the context of clinical immunotherapy/chemotherapy and maintenance treatment, using an aPD-1 with/without pemetrexed. To determine the optimal timing for administration of SKI-G-801, an AXL inhibitor, we investigated its anti-tumor effects based on inclusion at the immunochemotherapy and maintenance therapy stages. We also performed flow cytometry-based immune profiling of myeloid cells and lymphoid cells at different points in the treatment schedule, to investigate the immune activation and anti-tumor effects of the AXL inhibitor. The addition of SKI-G-801 to the immune checkpoint inhibitor and chemotherapy stage, as well as the maintenance therapy stage, produced the best anti-tumor results, and significant tumor growth inhibition was observed in both the TC1 and C3PQ models. Both models also exhibited increased proportion of effector memory helper T cells and increased expression of CD86^+^ macrophages. Especially, regulatory T cells were significantly reduced in the TC1 tumor model and there was an increase in central memory cytotoxic T cell infiltration and an increased proportion of macrophages with high CD80 expression in the C3PQ tumor model. These results suggest increased infiltration of T cells, consistent with previous studies using AXL inhibitors. It is expected that the results from this study will serve as a stepping stone for clinical research to improve the existing standard of care.

## Introduction

A recently developed lung cancer treatment strategy that combines an immune checkpoint inhibitor and chemotherapy has been applied as a standard treatment LUAD and LUSC, improving the outcomes of chemotherapy. Maintenance therapy with aPD-1 contributes to significantly improving the survival rate of patients by enhancing the effectiveness of immunochemotherapy and improving therapeutic efficacy. Nevertheless, lung cancer remains the leading cause of cancer-related mortality in men and women worldwide (1). The treatments for non-small cell lung cancer (NSCLC) (which accounts for the majority of lung cancers (2) include surgery, chemotherapy, targeted therapy, and recently developed immunotherapeutic agents. Among these treatments, chemotherapy is used as an adjuvant or primary therapy in cases of unresectable carcinoma or resistance to targeted therapies or immunotherapy (3). More than 70% of NSCLC patients present with advanced or metastatic cancer (stages 3-4) at the initial diagnosis (4). In cases where patients have stage 3 NSCLC which cannot be surgically resected, the combination of anti-PD-1 and platinum-based chemotherapeutic agents have shown promise, and an anti-PD-1 inhibitor and pemetrexed have been combined for extended treatment (5, 6). The synergistic effect of immunotherapy and chemotherapy has been reported to increase the immunogenicity of tumor cells by stimulating immunogenic cell death (ICD) (7, 8). ICD-induced apoptotic cells express high levels of non-mutated neoantigens and contribute to the priming of CD8^+^ T cells by antigen-presenting cells. In the study, Grimaldi et al. reported that T cells responded effectively to non-mutated neoantigens that resulted from a chemotherapy regimen which included cisplatin (CDDP) and anti-PD-1 therapy (9).

The AXL is expressed in tumor cells and plays an essential role in cancer resistance to chemotherapy and immunotherapy. Previous studies have verified the mechanism of action of a chemotherapy schedule that comprises AXL-targeted therapeutic agents combined with CDDP and pemetrexed, revealing that it plays a vital role in activating reactive oxygen species (ROS) (10). It has also been reported that AXL is closely associated with the process of epithelial-mesenchymal transition (EMT) in tumors (11). EMT signaling induced by AXL is a focus of targeted therapeutics (12), and it is associated with AXL-mediated suppression of MHC1 (HLA-A) expression, as well as increased PD-L1 levels, which is predicted to decrease the efficacy of immunotherapeutic agents *via* an AXL/PI3 kinase/PD-1 axis (13, 14). In studies on melanoma and lung adenocarcinoma, it has been reported that the function of the immune checkpoint inhibitor improved when AXL was targeted (11, 15).

Generally, LUSC has a poorer prognosis, usually occurs as a tumor confined to the proximal part of the upper airway, and is strongly associated with carcinogens such as tobacco smoke. On the other hand, LUAD is generally located in the periphery and occurs more frequently in nonsmokers, and driver oncogenes such as EGFR, ALK, and ROS are relatively high. In this study, TC1 cell lines generated by viral infection of primary epithelial cells showed similar tumorigenesis process to LUAD and were used as a tumor model for LUAD (16). C3PQ cell lines were utilized as a model for LUSC due to their similarity to LUSC in tumorigenic process and biochemical activity (double KRAS/WWOX; WW domain-containing oxidoreductase) (17). Thus, TC1 and C3PQ cell lines were treated with SKI-G-801, an AXL inhibitor, to investigate the efficacy of a chemo- and immunotherapy in order to prove potential effects of AXL inhibitor on therapeutic strategy and anti-tumor activity on LUSC and LUAD through a model-based analysis. By testing multiple treatment strategies (such as add-on or alternative therapy) against the existing standard of care (SoC), we were able to confirm that AXL inhibitors induce changes in the tumor microenvironment (TME), in addition to tumor suppression.

## Materials and Methods

### Reagents and Cell Lines

TC1 (CRL-2785), was purchased from American Type Culture Collection (ATCC; Manassas, VA, USA). C3PQ (Lacun-3) was kindly donated by Dr. Luis M. Montuenga (Center for Applied Medical Research (CIMA). All cells were cultured in RPMI 1640 (Corning, Tewksbury, NY, USA) supplemented with 10% fetal bovine serum (FBS) (ThermoFisher Scientific; Waltham, MA, USA) and 1% antibiotic-antimycotic (ThermoFisher Scientific; Waltham, MA, USA). Cells were grown in a humidified incubator at 37°C with 5% CO**_2_** and were tested regularly for mycoplasma contamination.

The AXL inhibitor SKI-G-801 was prepared as a 6 mg/ml solution in the recommended buffer (Sodium citrate, Citric acid, Sulfobutylether-β-Cyclodextrin in D.W. with pH 3). Paclitaxel was prepared as a 4 mg/ml solution in buffer (5% ethanol, 5% cremophor EL, 80% PBS) before injection. Pemetrexed, cisplatin, and carboplatin were prepared at 20 mg/ml, 1 mg/ml, and 10 mg/ml, respectively, in PBS before injection. Paclitaxel, pemetrexed, cisplatin, and carboplatin were purchased from Selleck Chemicals (Houston, TX, USA).

### Murine Tumor Models

All animal use and care protocols were reviewed and approved by the Institutional Animal Care and Use Committee of Avison Bio Medical Research Center in Yonsei University (IACUC number, 2019-0042). For the TC1 and C3PQ models, 7-week-old female C57BL/6 and BALB/c mice, respectively, were purchased from Orient Bio Inc. (Seongnam, South Korea). The mice were transferred to, established, and bred in an animal facility at Yonsei Medical College.

The animals were monitored for signs of toxicity over the course of treatments. Tumor volume and body weight were measured three times weekly using digital calipers and a scale. The bodyweights of TC1-engrafted C57BL/6 mice were recorded up to 30 days after treatment, to monitor toxicity. Tumor volume was calculated using the equation ‘V = length x width^2^ x 0.5’ (where ‘length’ = longest diameter and ‘width’ = shortest, perpendicular diameter) (18). The bodyweights of C3PQ-engrafted BALB/c mice were recorded up to 25 days after treatment.

For xenograft *in vivo* studies, 1×10^6^ TC1 cells were injected subcutaneously into the right flank of female C57BL/6 mice. For C3PQ, 5×10^6^ cells were injected subcutaneously into the right flank of female BALB/c mice. Tumor-bearing mice were randomly grouped based on an average tumor volume of approximately 100 mm^3^ for all treatment groups.

### Flow Cytometry

TC1 tumors were harvested from C57BL/6 mice on days 6, 10, and 16 after treatment initiation. C3PQ tumors were harvested from BALB/c mice on days 15 and 19 after treatment initiation, and tumors were processed for flow cytometry analysis. Tumors were enzymatically dissociated into single cells with collagenase type I (Worthington Biochemical, Lakewood, NJ, USA) for an hour at 37°C in shaker incubator (DAIHAN Scientific, Wonju, South Korea) and then filtered through a 70 μm cell strainer. Cells were washed with FACS buffer (PBS containing 1% BSA, 0.01% sodium azide, 0.5 mM EDTA) and blocked with FcR Blocking Reagent (Miltenyi Biotec, Bergisch Gladbach, Germany) at room temperature for 20 min. After fixation, cells were stained with True-Nuclear™ Transcription Factor buffer (Biolegend; San Diego, CA, USA) at room temperature for 30 min. Multi-color flow cytometry analysis was performed using a BD LSR-fortessa™ X-20 instrument (BD Bioscience; Franklin Lakes, NJ, USA). Mean fluorescence intensity (MFI) of dendritic cells and macrophages was measured by obtaining median. FlowJo software v10 (Tree Star; Ashland, OR, USA) was used for data acquisition and analysis.

### Immunohistochemistry

The numbers of tumor-infiltrating CD8+, CD3+, Foxp3+ T lymphocytes were measured by immunohistochemistry (IHC). The fluorescence image is a re-implemented mixed image of the DAB result obtained through a multispectral image analysis. All tumor tissue was analyzed, and 15 to 40 fields were analyzed per sample. The results were calculated by quantifying the number of total cells and the number of CD8+, CD3+, Foxp3+ cells per mm2. The whole slide scan and cell segmentation were performed in order to quantify the IHC results, and the degree of CD8+, CD3+, Foxp3+ total T lymphocytes infiltration was measured using the Vectra Polaris and InForm software. IHC was performed on the automatic staining machine, LEICA BOND RX. Digital images of IHC slides were obtained using a whole slide scanner. Image deconvolution was performed using the InForm software. The slides were stained with CD3e (CD3-12, Cell Signaling Technology; Beverly, MA, USA), CD8α (D4W2Z, Cell Signaling Technology; Beverly, MA, USA) and Foxp3 (D6O8R, Cell Signaling Technology; Beverly, MA, USA).

### Statistical Analysis

Data are reported as the mean ± SEM. Statistical analysis of tumor growth and survival data of TC1 and C3PQ mouse model were performed using analysis of variance (ANOVA) with the Mantel-Cox log-rank test of significance difference in GraphPad Prism (GraphPad Prism version 7.00 for Windows; GraphPad Software). Statistical analysis of flow cytometry was performed using a t-test in GraphPad Prism.

## Results

### The AXL Inhibitor, SKI-G-801, Significantly Enhances the Therapeutic Effects of a Chemo- and Immunotherapeutic Regimen on TC1 Tumor Model

**Figure 1:**
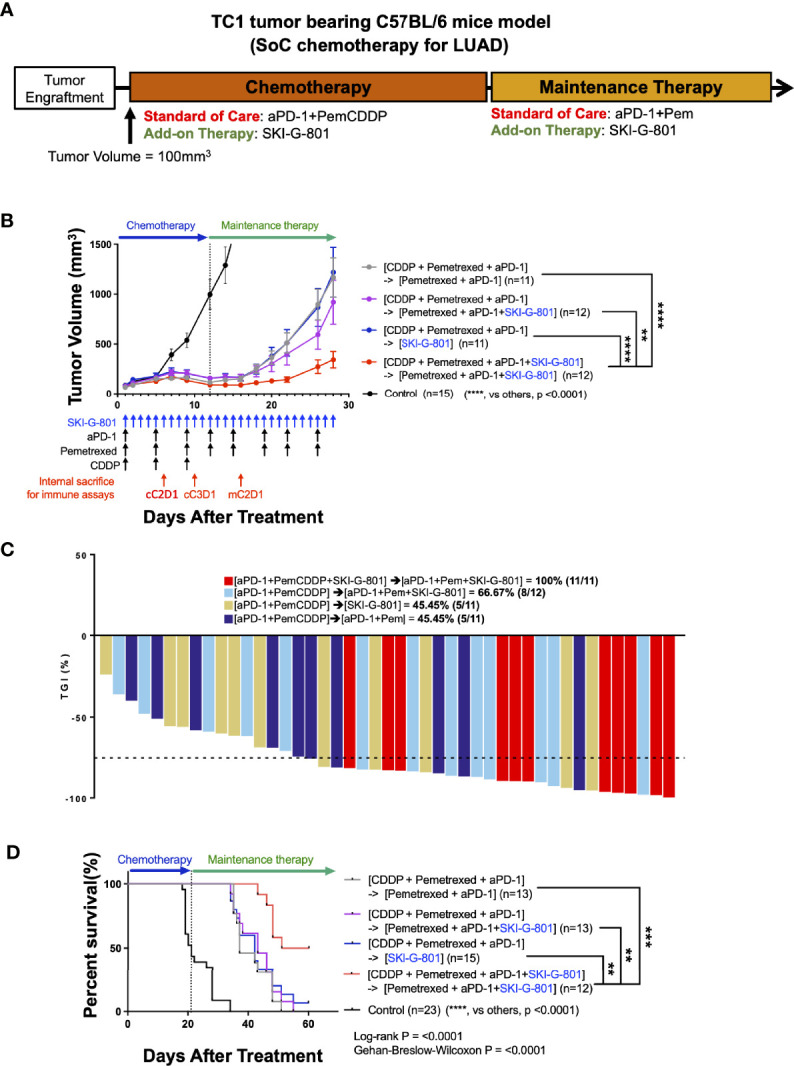
Upfront AXL inhibitor add-on delayed tumor growth, enhanced tumor growth inhibition, and improves survival in combination with SoC chemotherapy for LUAD in TC1 model **(A)** Syngeneic TC1 tumor cells were injected into C57BL/6 mice and treatment was initiated when the tumor volume reached 100 mm^3^. [aPD-1 + Pem + CDDP] were administered as standard of care for chemotherapy, and aPD-1 and Pem were administered as standard of care for maintenance therapy. **(B)** AXL inhibition by SKI-G-801 with standard of care significantly delayed the growth of TC1 xenografts in C57BL/6 mice. Red arrows indicate sampling points during the treatment schedule (SKI-G-801 30 mg/kg, QD; Pem 100 mg/kg, BIW; CDDP 5 mg/kg, BIW; aPD-1 10 mg/kg, BIW). Mean tumor volumes for each group and SEM images are shown. **(C)** Tumor growth inhibition was enhanced by upfront addition of SKI-G-801, with all of the mice in the [aPD-1+PemCDDP+SKI-G-801] → [aPD-1+Pem+SKI-G-801] group showing more than 75% TGI. Dashed line indicates -75% TGI. **(D)** Upfront AXL inhibition significantly enhanced the overall survival of TC1 tumor-bearing C57BL/6 mice. Statistical significance between groups was calculated using one-way ANOVA followed by Tukey’s multiple comparisons test (**p ≤ 0.01, ***p ≤ 0.001, ****p ≤ 0.0001). LUAD, lung adenocarcinoma; aPD-1, anti-PD-1 antibody; Pem, pemetrexed; CDDP, cisplatin; SEM, standard error of the mean; TGI, tumor growth inhibition; ANOVA, analysis of variance.

The standard treatment regimen for LUAD is cisplatin and pemetrexed with an anti-PD-1 inhibitor (6, 19). To investigate the therapeutic effect of the novel AXL kinase inhibitor, SKI-G-801, we performed an *in vivo* study with C57BL/6 mice bearing TC1 tumor (**Figure 1**). Treatment schedules started by initiating chemotherapy with cisplatin (CDDP), pemetrexed (Pem), and an immune checkpoint blockade for 3 cycles (Day + 12). After the initial regimen, treatment with anti-PD-1 and Pem was continued for maintenance therapy ([aPD-1+PemCDDP] → [aPD-1+Pem]) (**Figure 1A**). The effect of the AXL inhibitor SKI-G-801 was investigated as an add-on therapy from the beginning of treatment ([aPD-1+PemCDDP+SKI-G-801] → [aPD-1+Pem+SKI-G-801]), as an add-on at the maintenance stage ([aPD-1+PemCDDP] → [aPD-1+Pem+SKI-G-801]), or as a replacement for maintenance therapy ([aPD-1+PemCDDP] → [SKI-G-801]). Mice in the treatment group that included upfront addition of SKI-G-801 had the lowest tumor volumes (*vs*. [aPD-1+PemCDDP] → [aPD-1+Pem], p<0.0001; *vs*. [aPD-1+PemCDDP] → [aPD-1+Pem+SKI-G-801], p<0.01; *vs*. [aPD-1+PemCDDP] → [SKI-G-801], p<0.0001) (**Figure 1B**) and experienced dramatic inhibition of tumor growth (**Figure 1C**). The addition of SKI-G-801 to chemotherapy also enhanced overall survival in the TC1 tumor model. TC1 tumor-bearing mice which received vehicle injections had a median survival of 21 days after treatment, while mice receiving SoC treatment exhibited an extended median survival of 37 days (p<0.0001). However, treatment with upfront addition of SKI-G-801 significantly extended median survival to 55.5 days ([aPD-1+PemCDDP+SKI-G-801] → [aPD-1+Pem+SKI-G-801] *vs*. control, p<0.0001; *vs*. [aPD-1+PemCDDP] → [aPD-1+Pem], p<0.0001) (**Figure 1D**). Therefore, targeting AXL for inhibition is an approach worth considering in cancer therapy.

### AXL Inhibition Has Beneficial Therapeutic Effects in Combination in C3PQ Tumor Model

**Figure 2:**
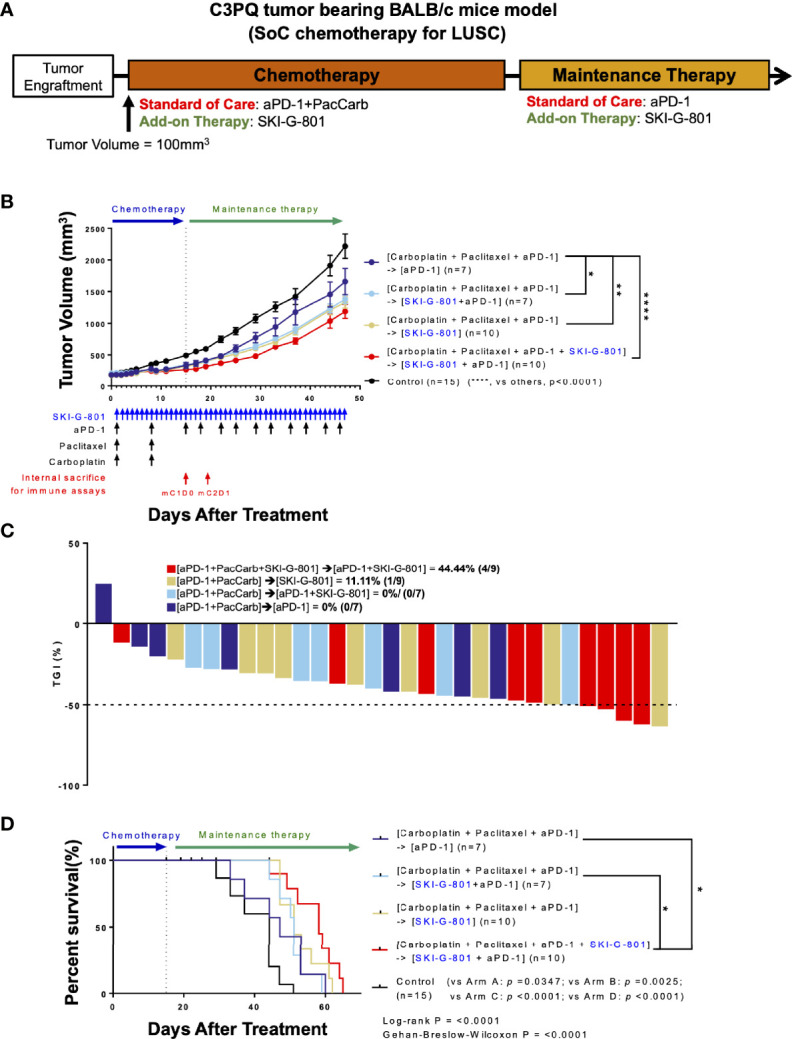
Upfront AXL inhibitor add-on delays tumor growth, enhances tumor growth inhibition, and survival in combination with SoC chemotherapy for LUSC in C3PQ model. **(A)** Syngeneic C3PQ tumor cells were injected into BALB/c mice and treatment was initiated when the tumor volume reached 100mm^3^. aPD-1 + Pac + Carb were administered as standard of care for chemotherapy, and aPD-1 was administered as standard of care for maintenance therapy. **(B)** AXL inhibition by SKI-G-801 with standard of care significantly delayed the growth of C3PQ xenografts in BALB/c mice. Red arrows indicate sampling points during the treatment schedule (SKI-G-801 30 mg/kg, QD; Pac 20 mg/kg, QW; Carb 50 mg/kg, QW; aPD-1 10 mg/kg, BIW). Mean tumor volumes of each group and SEM images are shown. **(C)** Tumor growth inhibition was enhanced by upfront addition of SKI-G-801, with all of the mice in the [aPD-1+PacCarb+SKI-G-801] → [aPD-1+SKI-G-801] group showing more than 50% TGI. Dashed line indicates -50% TGI. **(D)** Upfront AXL inhibition conferred enhanced overall survival to C3PQ tumor-bearing BALB/c mice. Statistical significance between groups was calculated using one-way ANOVA followed by Tukey’s multiple comparisons test (*p ≤ 0.05, **p ≤ 0.01, ****p ≤ 0.0001). LUSC, lung squamous cell carcinoma; aPD-1, anti-PD-1 antibody; Pac, paclitaxel; Carb, carboplatin; SEM, standard error of the mean; TGI, tumor growth inhibition; ANOVA, analysis of variance.

There are several different clinical subtypes of lung cancer, each of which has a different treatment regimen. Carboplatin (Carb) with paclitaxel (Pac) and anti-PD-1 is a well-known treatment for LUSC (5, 20). To determine whether AXL inhibition by SKI-G-801 enhances the therapeutic effect of this regimen, we performed an *in vivo* study with BALB/c mice bearing C3PQ tumors (**Figure 2**). The initial chemotherapy regimen comprised Carb, Pac, and an immune checkpoint blockade for 2 cycles (Day +16). Anti-PD-1 therapy was continued for maintenance therapy ([aPD-1+ PacCarb] → [aPD-1]) (**Figure 2A**). SKI-G-801 was included in the standard therapy regimen ([aPD-1+ PacCarb+SKI-G-801] → [aPD-1+SKI-G-801]), added during the maintenance therapy stage ([aPD-1+ PacCarb] → [aPD-1+SKI-G-801]), or used as a replacement for maintenance therapy ([aPD-1+ PacCarb] → [SKI-G-801]). Tumor-bearing mice that received SKI-G-801 had lower tumor volumes than mice on the standard treatment regimen ([aPD-1+ PacCarb] → [aPD-1] *vs*. [aPD-1+ PacCarb] → [aPD-1+SKI-G-801], p<0.05; *vs*. [aPD-1+ PacCarb] → [SKI-G-801], p<0.01; *vs*. [aPD-1+ PacCarb+SKI-G-801] → [aPD-1+SKI-G-801], p<0.0001) (**Figure 2B**). In addition, mice exposed to SKI-G-801 from the beginning of treatment showed greater inhibition of tumor growth (**Figure 2C**). AXL inhibition by SKI-G-801 also enhanced the overall survival of C3PQ tumor-bearing mice. Mice that received vehicle treatment had a median survival of 44 days, while the addition of the AXL inhibitor to the SoC regimen extended median survival to 58 days ([aPD-1+ PacCarb+SKI-G-801] → [aPD-1+SKI-G-801] *vs*. control, p<0.0001; *vs*. [aPD-1+ PacCarb] → [aPD-1], p<0.05; *vs* [aPD-1+ PacCarb] → [aPD-1+SKI-G-801], p<0.05) (**Figure 2D**). Taken together, these results indicate that AXL inhibition by SKI-G-801 with SoC treatment has significant benefits with respect to the inhibition of tumor growth and improved survival, in C3PQ tumor model.

### AXL Inhibition Enhances the Memory Function of the Immune System in Syngeneic Models

**Figure 3:**
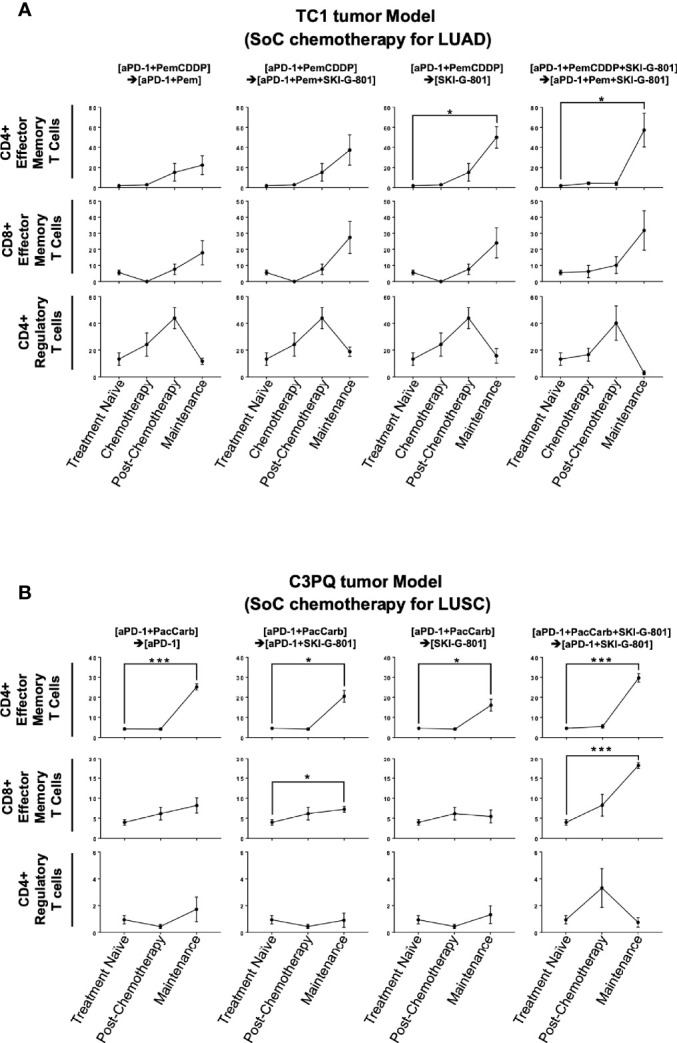
SKI-G-801 treatment increases memory function of the immune system. Analysis of T cell subset of TC1 tumor model **(A)** and C3PQ tumor model **(B)** by flow cytometry. **(A)** The effector memory subset of CD4^+^ T cells was significantly increased in the maintenance state of the [aPD-1+PemCDDP] → [SKI-G-801] group and the [aPD-1+PemCDDP+SKI-G-801] → [aPD-1+Pem+SKI-G-801] group compared to the treatment naïve group. The effector memory subset of CD8^+^ T cells showed an increase and CD4^+^ regulatory T cells showed a decreasing trend after chemotherapy, but these results were not significant. **(B)** The effector memory subset of CD4^+^ T cells was significantly increased in the maintenance state of all groups compared to the treatment naïve group. In addition, the effector memory subset of CD8^+^ T cells was increased in the maintenance state of the [aPD-1+PacCarb] → [aPD-1+SKI-G-801] group and the [aPD-1+PacCarb+SKI-G-801] → [aPD-1+SKI-G-801] group compared to the treatment naïve group. CD4^+^ regulatory T cells showed no meaningful changes. (*p ≤ 0.05, ***p ≤0.001).

As AXL is known to regulate the inflammatory response of the innate and adaptive immune systems (21), the mechanism of tumor growth inhibition may be related to these responses. Tumors were obtained at three or two different time points during the treatment schedule (**Figures 1B**, **2B**, respectively), and processed for use in flow cytometry to investigate immunodynamics after treatment. We compared four subsets of immune cells [CD8^+^ T cells, CD4^+^ T cells, dendritic cells (DCs), macrophages] between time points of internal sacrifice, each with a different treatment. Gating strategies were described in **Supplementary Figures 1A, B**. Overall, the results showed that as treatment continued, the effector memory subset of CD4^+^ T cells was increased by adding SKI-G-801 in both the TC1 and C3PQ tumor models compared to treatment-naive tumors (*p<0.05, ***p<0.001) (**Figures 3A, B**). Furthermore, in the C3PQ tumor model, the effector memory subset of CD8^+^ T cells was continuously increased by the initial inhibition of AXL (***p<0.001) (**Figure 3B**). Similarly, in the TC1 model, an increase of CD8+ effector memory T cells was confirmed by treating SKI-G-801, but it was not significant (**Figure 3A**). Although the number of regulatory T cells in the TC1 model showed a decreasing in the maintenance phase of the group initially added with SKI-G-801, it was not significantly improved by SKI-G-801 as a whole (**Figures 3A, B**).

**Figure 4:**
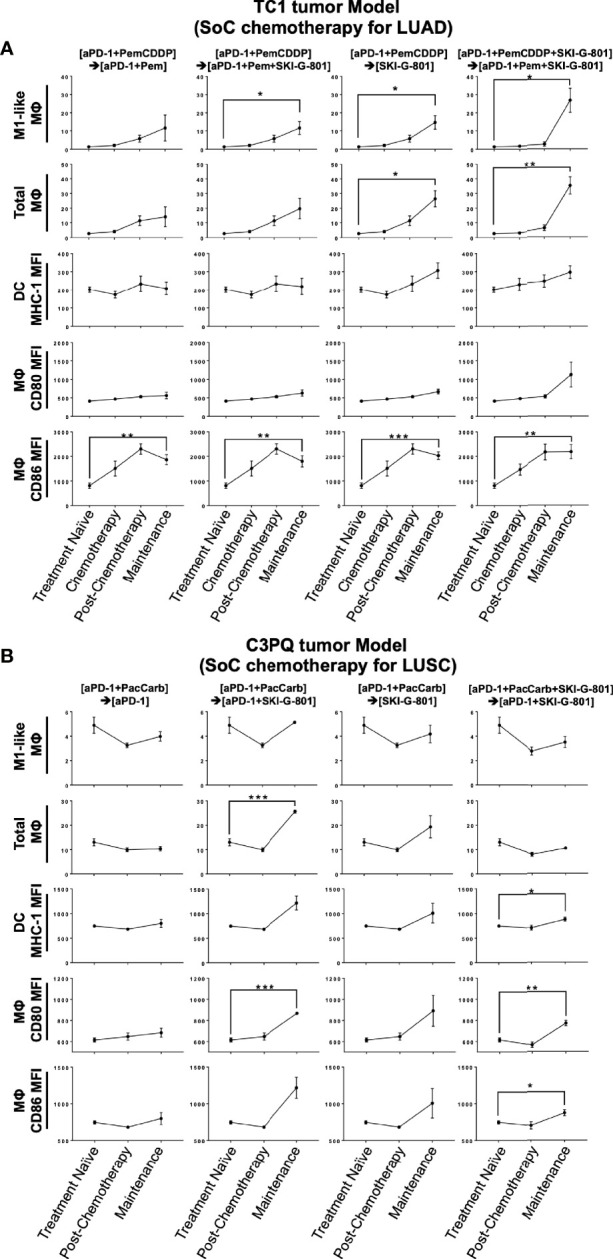
Antigen-presenting machinery increases after SKI-G-801 treatment. Analysis of myeloid cell subset of TC1 tumor model **(A)** and C3PQ tumor **(B)** by flow cytometry. **(A)** M1-like macrophages were significantly increased in the maintenance state of the [aPD-1+PemCDDP] → [aPD-1+Pem+SKI-G-801] group, the [aPD-1+PemCDDP] → [SKI-G-801] group, and the [aPD-1+PemCDDP+SKI-G-801] → [aPD-1+Pem+SKI-G-801] group compared to the treatment naïve group. The macrophage total also increased in the maintenance state of the [aPD-1+PemCDDP] → [SKI-G-801] group and the [aPD-1+PemCDDP+SKI-G-801] → [aPD-1+Pem+SKI-G-801] group compared to the treatment naïve group. The number of CD86 expressing macrophages was significantly high in all groups compared to the treatment naïve group. **(B)** M1-like macrophages showed a decreasing trend in all groups, but total macrophage count was significantly increased in the maintenance state of the [aPD-1+PacCarb] → [aPD-1+SKI-G-801] group compared to the treatment naïve group. In addition, the number of CD86 and CD80 expressing macrophages increased in the maintenance state of the [aPD-1+PacCarb] → [aPD-1+SKI-G-801] group and the [aPD-1+PacCarb+SKI-G-801] → [aPD-1+SKI-G-801] group compared to treatment naïve state. (*p ≤ 0.05, **p ≤ 0.01, ***p ≤0.001).

Flow cytometry analysis of myeloid cells showed that SKI-G-801 treatment increased the number of M1-like macrophages (F4/80^+^CD206^+^CD11c^-^) and total macrophages (CD45^+^F4/80^+^) in the TC1 tumor model (*p<0.05, **p<0.01) (**Figure 4A**). Among antigen-presenting cells, macrophages (F4/80^-^) and DCs (CD11c^+^F4/80^-^) also displayed a corresponding increase. Especially, mean fluorescence intensity (MFI) of CD86^+^ and CD80^+^ macrophages were increased after SKI-G-801 treatment in TC1 model (**p<0.01, ***p<0.001) (**Figure 4A**). On the other hand, MFI of MHC-1^+^ DCs showed no significant change. As similar with TC1 model, the MFI of CD86^+^ macrophages was significantly increased, but CD80^+^ macrophages did not significantly increase after SKI-G-801 treatment in C3PQ model (*p<0.05, **p<0.01, ***p<0.001). In addition, the MFI of MHC-1^+^ DCs was increased in upfront treatment of SKI-G-801 (*p<0.05) (**Figure 4B**). However, there were no significant change in the number of M1-like and total macrophages but only [aPD-1+ PacCarb] → [aPD-1+SKI-G-801] group were significantly increased in the number of total macrophages (***p<0.001) (**Figure 4B**). Not only TC1 model but also C3PQ model showed increase of CD4^+^ effector memory T cells upfront treatment of SKI-G-801 compared to other groups however, only CD8^+^ effector memory T cells were increased in C3PQ model. Moreover, regulatory T cells were decreased in upfront treatment of SKI-G-801 compared to other groups only for TC1 model (*p<0.05, **p<0.01) (**Supplementary Figure 2A**). Macrophages also showed an overall increase in the TC1 model but were not consistent in the C3PQ model (*p<0.05) (**Supplementary Figure 2B**). Upfront AXL inhibition in the TC1 model reduced the proportion of regulatory T cells. In the C3PQ model, the numbers of CD8^+^ central memory cells and CD80^+^ macrophages increased. The effector memory of CD4^+^ T cells and the amount and CD80 expression of macrophages were enhanced in both the TC1 and C3PQ tumor models.

**Figure 5:**
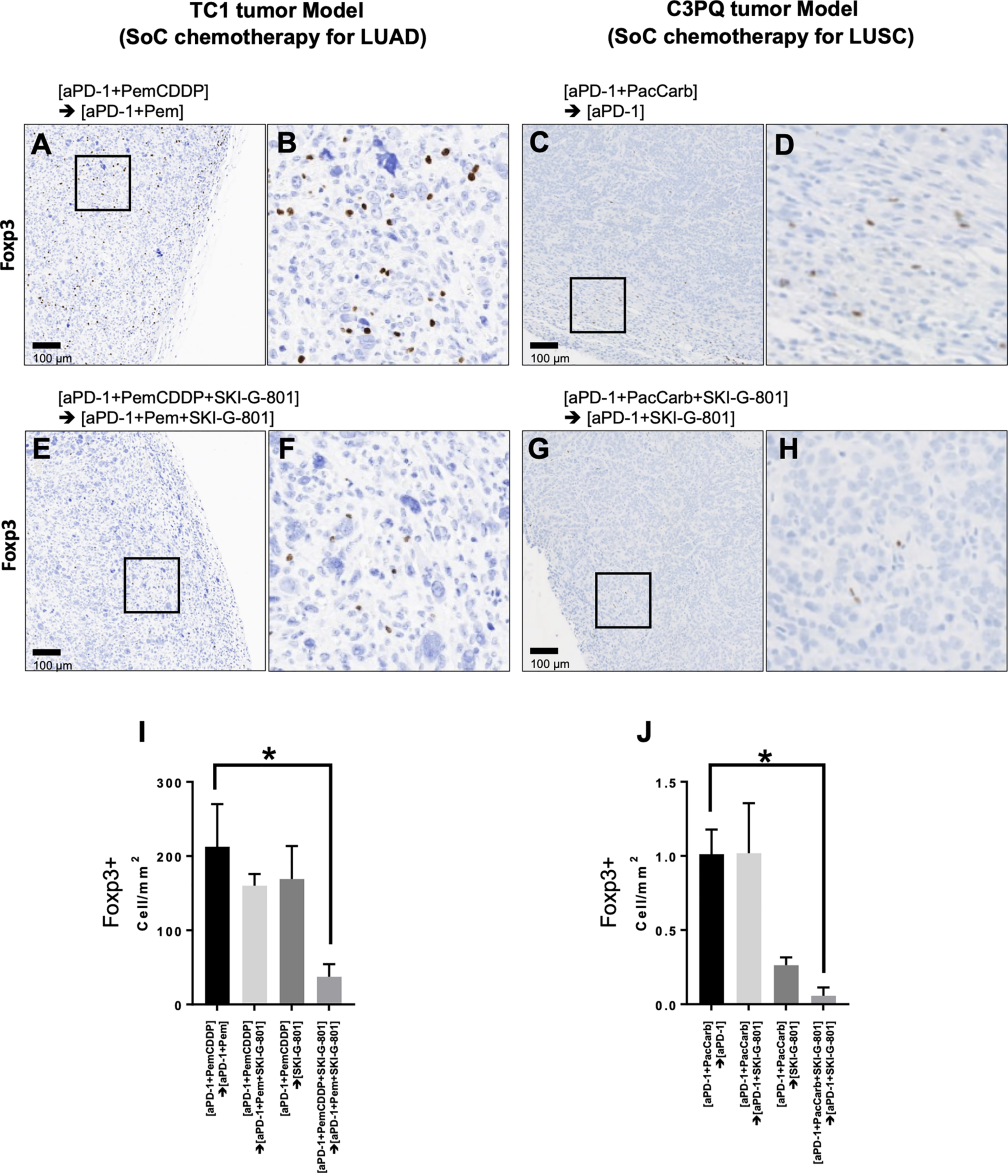
Immunohistochemistry with anti-Foxp3 reveals tumor infiltration by regulatory T cells. Positive Foxp3 expression was observed in the [aPD-1+PemCDDP] → [aPD-1+Pem] **(A)** and [aPD-1+PemCDDP+SKI-G-801] → [aPD-1+Pem+SKI-G-801] **(E)** groups in TC1 tumor tissues. **(B)**, Higher magnification of images in **(A, F)**, higher magnification of images in **(E)**. In addition, positive Foxp3 expression was visible in the [aPD-1+ PacCarb] → [aPD-1] **(C)** and [aPD-1+ PacCarb+SKI-G-801] → [aPD-1+SKI-G-801] **(G)** groups in C3PQ tumor tissues. **(D)**, Higher magnification of images in **(C, H)**, higher magnification of images in **(G)**. **(I)**, the number of Foxp3^+^ cells per mm^2^ of TC1 tumor tissues and **(J)**, the number of Foxp3^+^ cells per mm^2^ of C3PQ tumor tissues. *p < 0.05. Scale bars in **(A, C, E, G)**: 100µm. (*p ≤ 0.05).

Anticancer immune regulation is a critical factor that contributes to successful cancer treatment. Foxp3^+^CD4^+^ regulatory T cells (Tregs) did not show significant changes in response to treatment schedule in flow cytometry analysis (**Figures 3A, B**). However, immunohistochemistry revealed that AXL inhibition reduced the number of Foxp3^+^ Tregs (cells/mm^2^) that infiltrated tumor tissues in both the TC1 and C3PQ tumor models (*p<0.05) (**Figure 5**). We also confirmed the immunodynamics in the immunohistochemistry. There were no significant changes in CD3^+^, CD8^+^ T cells in TC1 and C3PQ tumor model. However, in TC1 model, there were significantly increased of Foxp3^+^ T cells but not in upfront treatment of SKI-G-801 group (**Supplementary Figure 3**).

**Figure 6:**
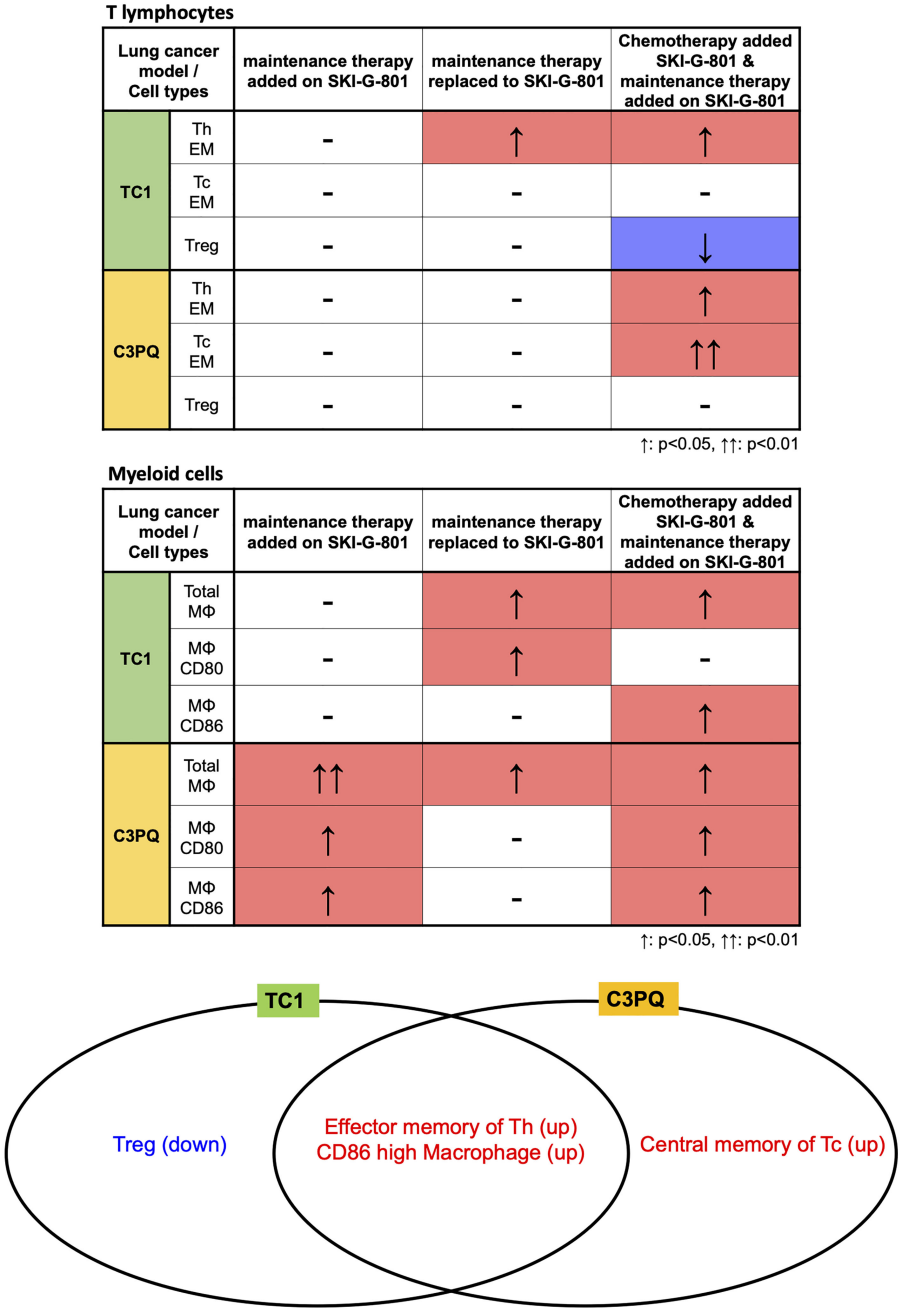
Immunological benefits of SKI-G-801 addition to chemotherapies in TC1 and C3PQ tumor models. The differences in immune cell changes between TC1 and C3PQ after treatment in each group is shown. The number of effector memory Th cells and CD86^+^ high macrophages was similar in TC1 and C3PQ. In particular, Tregs were downregulated in TC1, and central memory of Tc cells were upregulated in C3PQ. ↑: p < 0.05, ↑↑: p < 0.01.

Figure summarizing the immunological benefits of SKI-G-801 administration as part of the chemotherapy regimens demonstrate a common advantage of this inhibitor, in the form of enhanced CD4^+^ effector memory cells and increased macrophages with a high expression of CD86, in both TC1 and C3PQ tumor models (**Figure 6**). However, there were some factors that distinguished the two syngeneic models. AXL inhibition resulted in a dramatic suppression of Tregs in the TC1 tumor model (**Figures 5**, **6**) but produced a less effective or not significant effect in the C3PQ model (**Figures 5**, **6**). The increase in CD8^+^ central memory T cells was significant only in the C3PQ tumor model with upfront inhibition of AXL. Taken together, the effects of improving antitumor activity and activating immune cells in combination with chemotherapy and aPD-1 were the best in the continuous treatment of SKI-G-801.

## Discussion

In this study, our strategy for testing the efficacy of the AXL inhibitor, SKI-G-801, by administration at different stages of the existing SoC protocol revealed that the most effective treatment method was the addition of SKI-G-801 at the chemotherapy stage, although add-on treatment at the maintenance therapy stage also proved to be effective. Lung cancer models derived from mice and the analysis of TC-1 and C3PQ cell lines should be used as a supplementary data to clinical LUAD or LUSC lung cancer studies. In this study, SoC chemotherapy for LUAD and LUSC was applied to TC1 and C3PQ cell lines that were showing similar carcinogenic properties with LUAD and LUSC, respectively. It is difficult to say that it is an optimized model for evaluation of chemotherapy or immunotherapy for clinical studies or treatments. Nevertheless, treatment response to combination or maintenance therapy of SKI-G-801 is useful to understand the general immune response in cell lines that closely mimic NSCLC lung cancer. Furthermore, these strategies were effective in both the TC1 tumor model treated with LUAD treatment strategy and C3PQ tumor model treated with LUSC treatment strategy. Although we hypothesized that combined treatment using an AXL inhibitor could cause toxicity in addition to that associated with the existing platinum chemotherapy, our data indicated that the toxicity to SKI-G-801 itself was not high, and we did not observe either weight or behavioral changes in mice subjected to combination treatments (**Supplementary Figure 4**). In our TC1 model results, body weight was observed to drop during chemotherapy as similar with previous study, but no mice died until the entire treatment strategy was progressed. In addition, it was confirmed that body weight recovered from the time when cisplatin administration was terminated and SKI-G-801 was administered. Individual studies have reported the efficacy of immunotherapeutic agents for AXL combination therapy (22), targeted therapies (23), and increased anticancer effects on chemotherapy (24). A preclinical study investigating AXL, and DNA damage showed that AXL can effectively improve chemotherapy, by accumulation of the DNA damage markers 53BP1 and RAD51 in tumors (25). ONO-7475, an AXL inhibitor developed by ONO, improved the therapeutic effect of osimertinib (a third-generation EGFR-TKI) and overcame resistance in the osimertinib-resistant PC-9 and HCC4011 cell lines (26). In addition, AXL inhibitors have demonstrated efficacy alongside anti-tumor vaccines. In particular, TAM receptors were highly expressed in the monocyte-derived IL-10^+^DC induced by the vaccine, and in a preclinical model, inclusion of an AXL inhibitor increased the immune response of the vaccine by regulating the expression of the TAM receptors (27). However, none of these previously published studies have described preclinical experiments that demonstrate the potential for improvement over the standard treatment methods currently applied in clinical practice, as well as a comprehensive analysis of the immune response.

In the current study, the effect of the AXL inhibitor on immune cells in the tumor microenvironment was demonstrated in two tumor types with different characteristics. In-depth characterization of immune cell dynamics was performed by analyzing samples at each treatment step using flow cytometry to confirm the proportion and response of the immune cells. In both TC1 and C3PQ tumors, increases in macrophages with high expression of CD86 (a cofactor involved in antigen presentation) and effector memory helper T cells were common features of the treatment protocols that provided the most beneficial outcomes. As similar with previous report (28), there were decrease of Treg, which play an important role in immunosuppression, were significantly reduced In the TC1 tumor model. While in the C3PQ tumor model, central memory cytotoxic T cell infiltration was increased and the ratio of macrophages expressing CD80 was high. Several of the existing AXL studies have also noted substantial T cell infiltration and long-term memory (29), but in contrast to previous reports, we did not observe a significant increase in the ratio of DCs.

The increase in CD86^+^ in antigen-presenting cells is another result that is consistent with previous studies (**Figure 5**) (29). These differences are considered to be due to differences in the immune cells infiltrating the tissues based on the carcinoma. However, we confirmed that the increase in antigen-presenting machinery due to AXL inhibition is a common mechanism of action. IHC analysis also confirmed a decrease in Tregs in both carcinomas, in both upfront add-on therapy and maintenance add-on of the AXL inhibitor. No previous studies have shown a direct association between AXL and Tregs. It was known that TGF-β and epithelial-mesenchymal transition can promote the priming of induced Tregs in the tumor microenvironment (27). However, there is still no direct evidence regarding Treg regulation by AXL and further studies are needed to clarify this point. When we analyzed the changes in the various immune cell subsets (CD4^+^, CD8^+^, Treg, macrophage, DC, etc.) using an immunodynamic approach, we observed that the addition of SKI-G-801 to the chemotherapy and extended therapy SoC regimens produced dramatic effects. For example, the proportion of effector memory Th cells is quite high during extension treatment, which explains the clinical reason for the extension treatment outcomes. The benefits of adding AXL inhibition to the SoC strategy is clear when one examines the level of priming induced by the AXL inhibitor. The T cell responses were the same regardless of the type of carcinoma, whereas the myeloid cell responses were different. In the TC1 tumor model, M1 macrophages were significantly increased by AXL inhibition, with a sharp increase compared to the SoC protocol. This is likely related to the infiltration of T cells and is expected to result in an increase in chemokines in the tumor tissue. On the other hand, in the C3PQ tumor model, we hypothesize that the function of APC was improved by an increase in DC and macrophage co-stimulating factors.

As of 2021, there are approximately 60 clinical studies underway worldwide focused on anticancer drug research targeting AXL, and various clinical studies have already been conducted to investigate chemotherapy combinations, immunotherapy combinations, adjuvant-neoadjuvant treatment strategies, and combination treatment strategies with TKIs. In line with these trends, this translational study was aimed at improving the current chemotherapy and ICI combination therapy protocol, by observing the proportion and changes in immune cells using an immunodynamic approach that cannot be applied during clinical treatment of patients.

## Data Availability Statement

The original contributions presented in the study are included in the article/**Supplementary Material**. Further inquiries can be directed to the corresponding author.

## Ethics Statement

The animal study was reviewed and approved by Yonsei University IACUC 2019-0042.

## Author Contributions

K-HP, D-SJ, SP, and HL: conceptualization. WL, DK, C-BS, and YC: formal analysis. WL and DK: writing—original draft. WL, DK, JK, and YB: visualization. YK, SL, SYL, YJ, EL, MY, SH, WY, JJ, and EK: investigation and data curation. K-HP, JP, JDP, DL, and H-WK: validation. K-HP, SML, MH, B-CA, and JL: resources and supervision. All authors contributed to the article and approved the submitted version.

## Conflict of Interest

Authors WL and MY were employed by JEUK Co., Ltd and HL, SP, D-SJ, and YC were employed by Oscotec Inc.

The remaining authors declare that the research was conducted in the absence of any commercial or financial relationships that could be construed as a potential conflict of interest.

## Publisher’s Note

All claims expressed in this article are solely those of the authors and do not necessarily represent those of their affiliated organizations, or those of the publisher, the editors and the reviewers. Any product that may be evaluated in this article, or claim that may be made by its manufacturer, is not guaranteed or endorsed by the publisher.

## Supplementary Material

Supplementary Figure 1 Gating strategy for flow cytometry. **(A)** T cell subset, **(B)** Myeloid cell subset.

Supplementary Figure 2 Analysis of T cell **(A)** and myeloid cell **(B)** subsets of TC1 and C3PQ tumor models by flow cytometry. CD4+ effector memory T cells were increased in the [aPD-1+PemCDDP+SKI-G-801] → [aPD-1+Pem+SKI-G-801] group in the TC1 model, and in the [aPD-1+ PacCarb+SKI-G-801] → [aPD-1+SKI-G-801] group in the C3PQ model, compared to the [aPD-1+chemo] → [aPD-1] group of each model. Specifically, CD8+ effector memory T cells showed an increase only in the C3PQ model and CD4+ regulatory T cells showed a decrease only in the TC1 model. However, CD86 and CD80 expressing macrophages and the total macrophage count increased after treatment with SKI-G-801 in both models.

Supplementary Figure 3 Immunohistochemistry with anti-Foxp3, anti-CD3, and anti-CD8 for measurement of tumor infiltrating T lymphocytes. The number of positive Foxp3 cells was significantly increased after treatment with SKI-G-801 only in the TC1 model, but not in the [aPD-1+PemCDDP+SKI-G-801] → [aPD-1+Pem+SKI-G-801] group.

Supplementary Figure 4 Body weight of TC1 tumor model **(A)** and C3PQ tumor model **(B)**.
