# Supplementary figures and images for: Incorporation of SKI-G-801, a Novel AXL Inhibitor, With Anti-PD-1 Plus Chemotherapy Improves Anti-Tumor Activity and Survival by Enhancing T Cell Immunity
Source: Front Oncol. 2022 Mar 9;12:821391. doi: 10.3389/fonc.2022.821391 (PMC8959645; doi:10.3389/fonc.2022.821391)

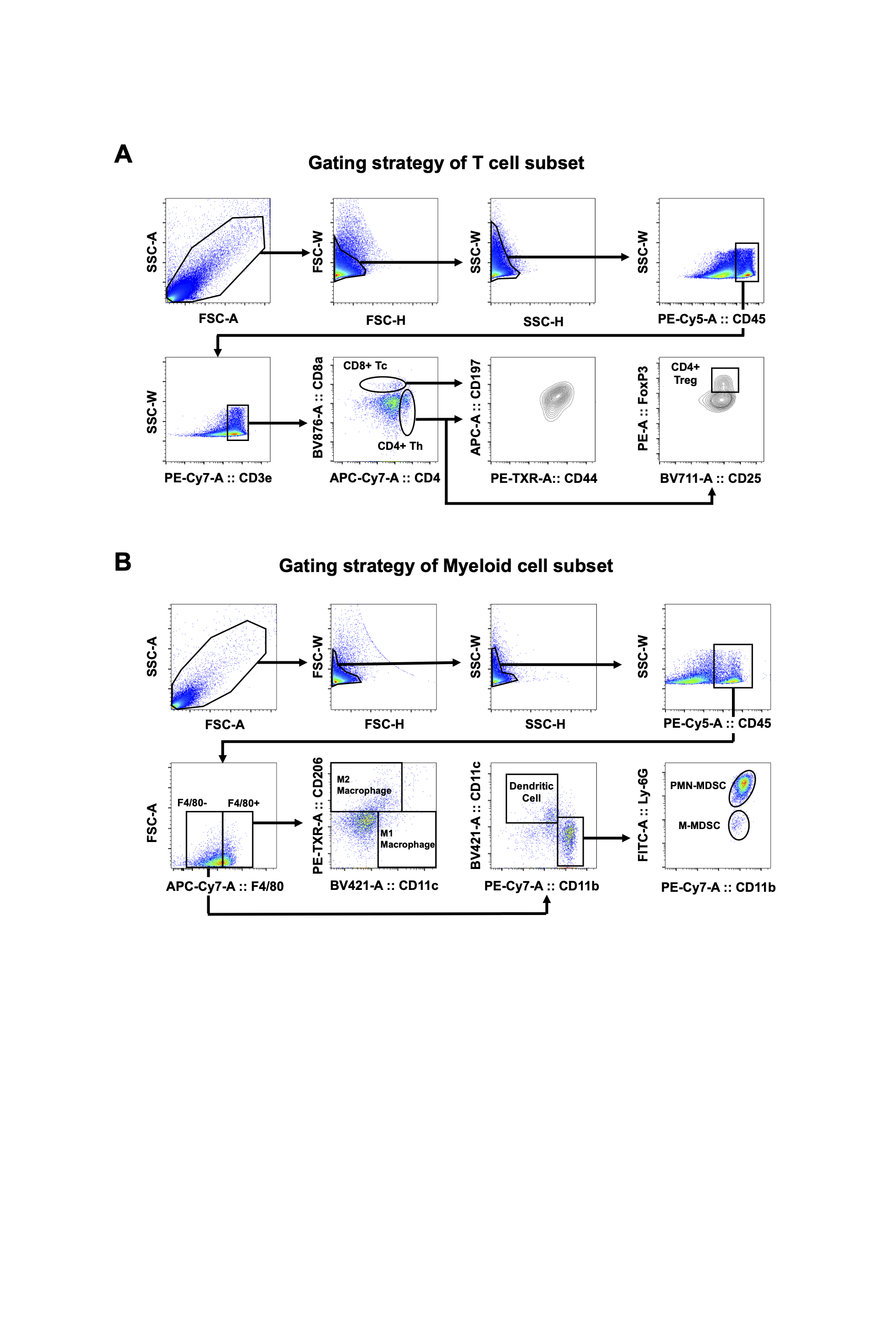

Supplement: Supplementary Figure 1 — Gating strategy for flow cytometry. (A) T cell subset, (B) Myeloid cell subset. [file Image_1.tiff]

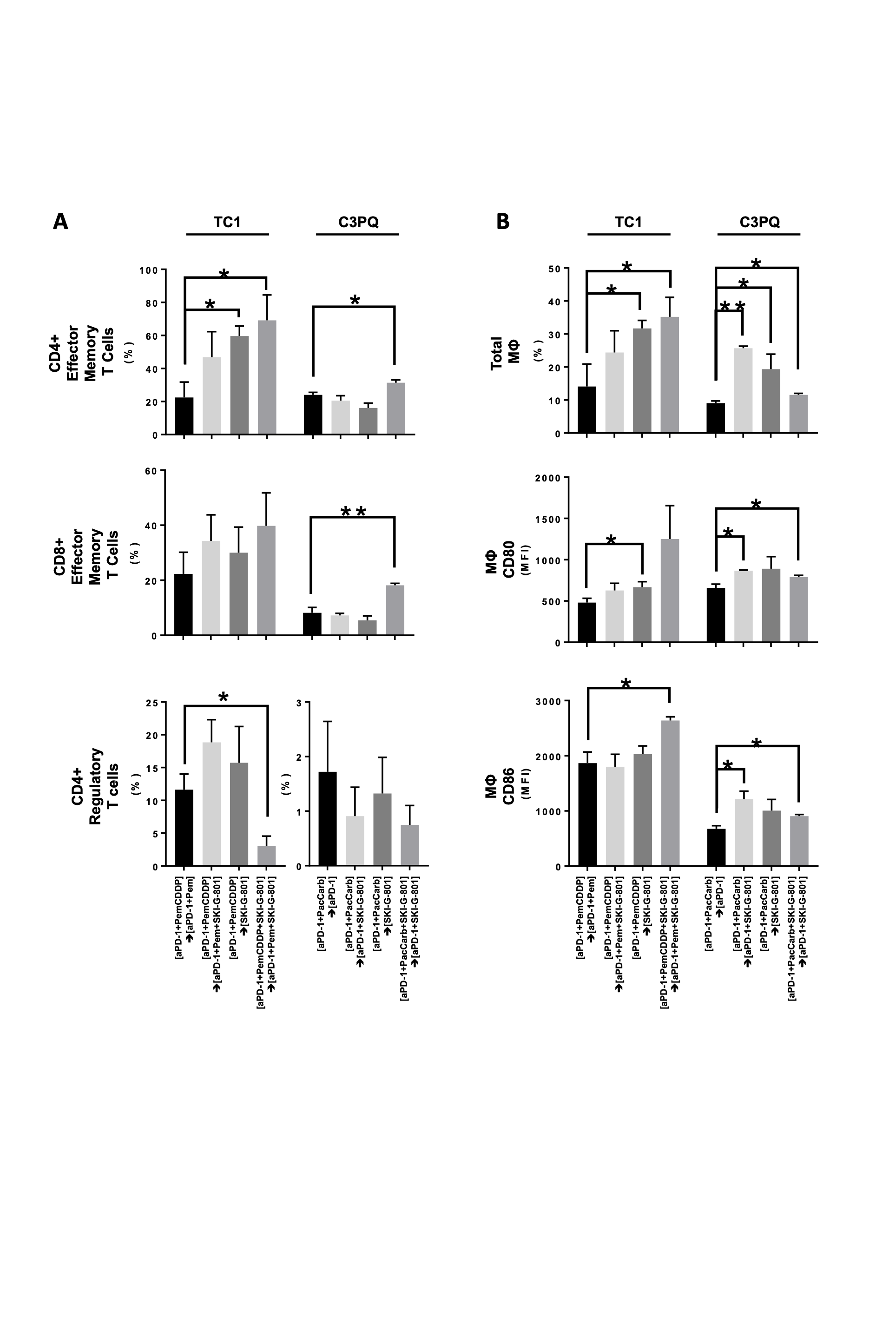

Supplement: Supplementary Figure 2 — Analysis of T cell (A) and myeloid cell (B) subsets of TC1 and C3PQ tumor models by flow cytometry. CD4+ effector memory T cells were increased in the [aPD-1+PemCDDP+SKI-G-801] → [aPD-1+Pem+SKI-G-801] group in the TC1 model, and in the [aPD-1+ PacCarb+SKI-G-801] → [aPD-1+SKI-G-801] group in the C3PQ model, compared to the [aPD-1+chemo] → [aPD-1] group of each model. Specifically, CD8+ effector memory T cells showed an increase only in the C3PQ model and CD4+ regulatory T cells showed a decrease only in the TC1 model. However, CD86 and CD80 expressing macrophages and the total macrophage count increased after treatment with SKI-G-801 in both models. [file Image_2.tiff]

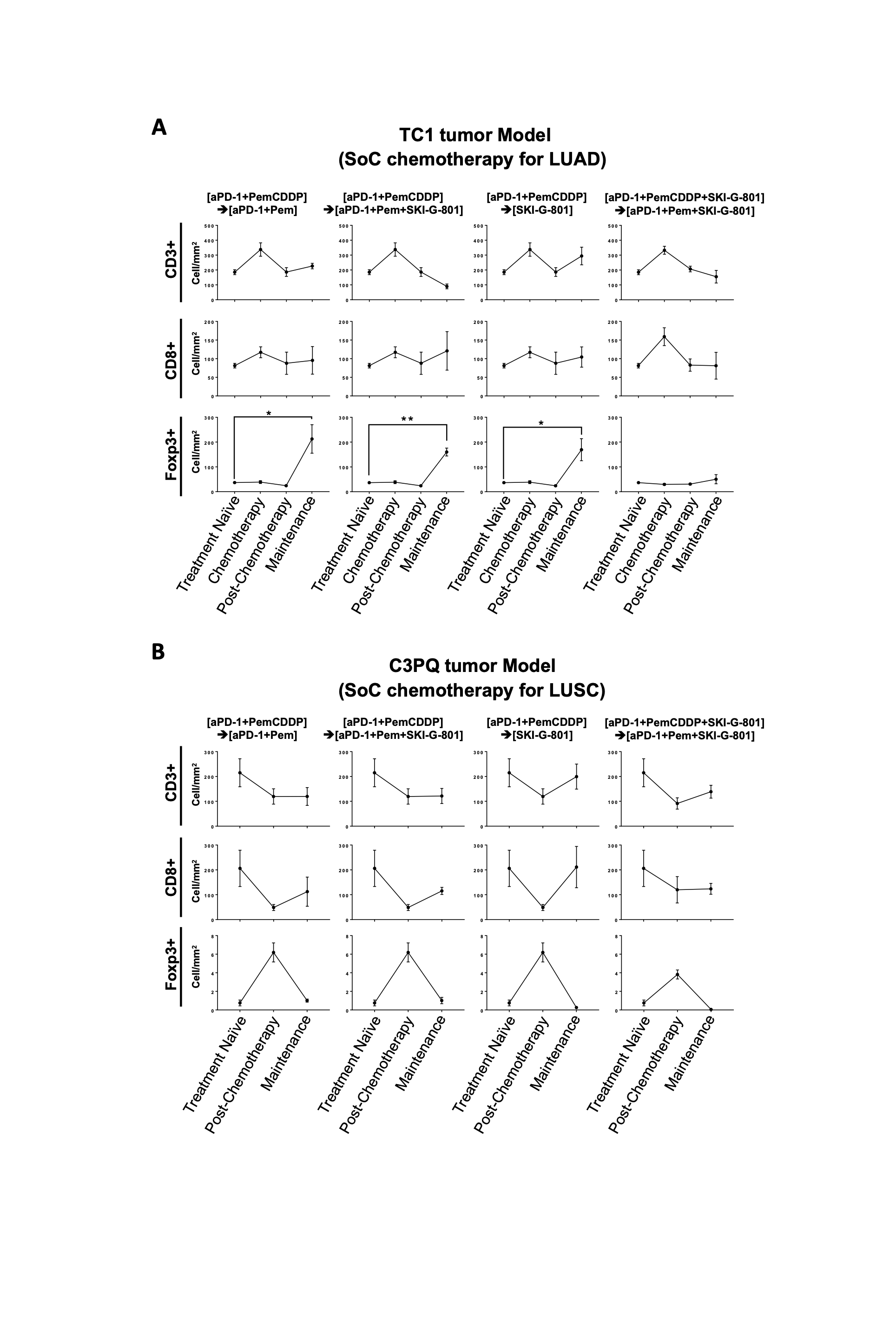

Supplement: Supplementary Figure 3 — Immunohistochemistry with anti-Foxp3, anti-CD3, and anti-CD8 for measurement of tumor infiltrating T lymphocytes. The number of positive Foxp3 cells was significantly increased after treatment with SKI-G-801 only in the TC1 model, but not in the [aPD-1+PemCDDP+SKI-G-801] → [aPD-1+Pem+SKI-G-801] group. [file Image_3.tiff]

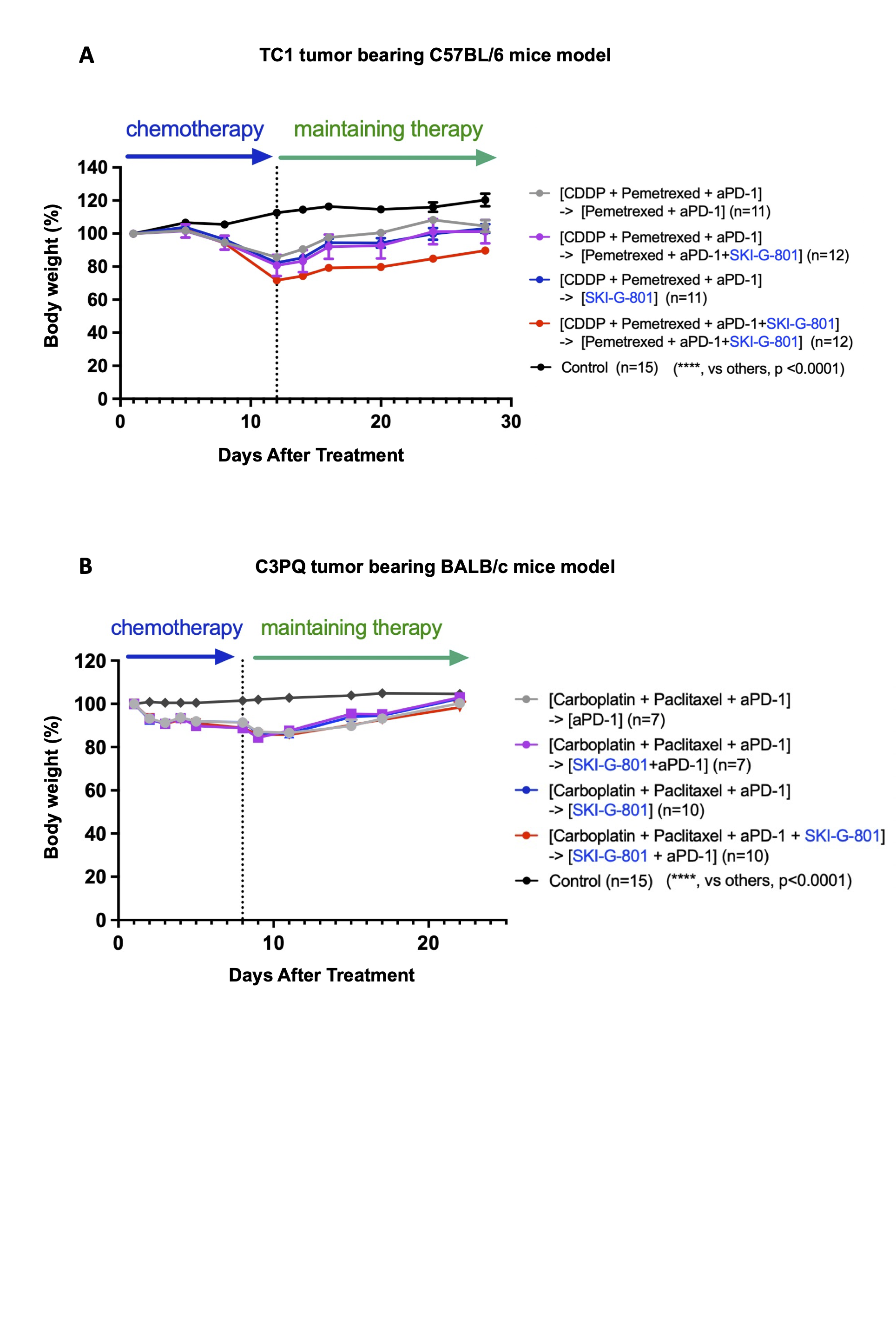

Supplement: Supplementary Figure 4 — Body weight of TC1 tumor model (A) and C3PQ tumor model (B). [file Image_4.tiff]
